# The associations between intensive mothering beliefs, maternal overparenting behavior and Chinese preschoolers' behavioral problems

**DOI:** 10.3389/fpsyg.2026.1814185

**Published:** 2026-05-08

**Authors:** Xiaoying Xia

**Affiliations:** School of Education, Shanghai Normal University Tianhua College, Shanghai, China

**Keywords:** intensive mothering beliefs, overparenting behavior, internalizing problems, externalizing problems, Chinese preschoolers

## Abstract

**Introduction:**

Intensive mothering (IM) has become the prevalent motherhood ideology in many societies, however, the current understanding of how IM beliefs are associated with young children's development outcomes remains limited.

**Methods:**

The present study examined the associations between IM beliefs, maternal overparenting behavior, and Chinese preschoolers' behavioral problems. A sample of 1,025 Chinese mothers (*Mean age* = 36.42 years, *SD* = 3.42) and their children (*Mean age* = 4.94 years, *SD* = 0.72) from eight kindergartens in Shanghai, China participated in this study.

**Results and Discussion:**

Results showed that mothers' endorsement of essentialist, challenging, and child-centered IM beliefs accounted for significant variances in children's internalizing and externalizing behaviors. In addition, stronger endorsement of IM beliefs related to higher maternal overparenting and overparenting behavior partially accounted for the associations between IM beliefs and children's behavioral problems. These findings highlight how prevalent intensive mothering ideologies may shape parenting practices and children's social-emotional adjustment in early childhood. Implications for family professionals and early childhood practitioners were discussed.

## 1 Introduction

The preschool years represent a critical period for children to acquire social-emotional skills. Social-emotional and behavioral problems that emerge in the preschool stage are consistent predictors of maladjustment in adolescents and adulthood (Korhonen et al., 2018). Children's behavior problems can generally be classified into internalizing (e.g., social withdrawal, anxiety, and depressive symptoms) and externalizing (e.g., aggression, hyperactivity, impulsivity) problems (Achenbach et al., 1991). In early years, children's lives are mostly centered around their immediate family, and parenting beliefs and practices have a critical role in shaping young children's social-emotional adjustment (Bornstein et al., 2018).

Over the past few decades, child-rearing has become increasingly intensive and child-centered. Hays (1996) attributed this trend to intensive mothering (IM), a constellation of beliefs about appropriate caregiving for children and maternal behaviors. Central to this set of beliefs, mothers are generally positioned as the primary caregivers and are expected to devote substantial time, energy, and financial resources to ensure children's optimal development and future success. Existing research indicates that distinct facets of IM have a mix of positive, negative and null relationships with children's motor, language and social development outcomes (Egami, 2024; Schiffrin et al., 2015). Hence, further empirical work is needed to clarify how multiple dimensions of IM beliefs relate to children's development outcomes.

Bornstein et al. (2018) proposed that parental cognitions (i.e., childrearing beliefs, attitudes and knowledge) shape their parenting behaviors and practices, which in turn affect children's adjustment. A growing body of research suggests that IM beliefs are linked to overparenting behaviors such as anticipatory problem solving, overprotection and excessive involvement (Schiffrin et al., 2015; Turner et al., 2023). Overparenting is understood as developmentally inappropriate parenting practices that far exceed children's actual needs (Segrin et al., 2012). Parents who overparent their children tend to give excessive advice, offer unnecessary assistance, and preemptively solve problems on behalf of their children (Segrin et al., 2012). Prior studies have linked overparenting to a range of negative outcomes, including higher behavioral problems (Rousseau and Scharf, 2015) and difficulties in interpersonal relationships (Segrin et al., 2015). Nevertheless, the current literature has mostly sampled adolescents or emerging adults in the Western contexts and the effects of overparenting on young children's development outcomes in non-Western contexts remain underexplored. China exemplifies the country where intensive parenting has become the prevailing norm (Doepke and Zilibotti, 2019). Chinese traditional values emphasize parental responsibility for children's success and mothers typically bear primary responsibilities for child-rearing. However, little empirical work has examined how IM beliefs and overparenting behaviors jointly shape young children's development and adjustment. Therefore, the present study aimed to examine the associations between IM beliefs, maternal overparenting behaviors, and preschoolers' behavior problems within the Chinese cultural context.

### 1.1 Intensive mothering beliefs

The concept of intensive mothering (IM) was first proposed by Hays (1996) to describe the prevailing Western parenting model in which mothers are the preferred primary caregivers and parenting tend to be “child-centered, expert-guided, emotionally absorbing, labor-intensive, and financially demanding” (Hays, 1996, p.8). Recent studies indicates that intensive mothering has emerged as the dominant parenting discourse in many societies and has gained widespread acceptance across racial and socioeconomic groups (Lubiewska et al., 2025). Building on Hays' original conceptualization, Liss et al. (2013) operationalized IM as a multidimensional belief system and identified five core components: essentialism, fulfillment, stimulation, challenging and child-centered. Specifically, essentialism reflects the belief that mothers are inherently more capable and better suited for care-giving than fathers. Fulfillment belief describes parenting as a fulfilling and rewarding endeavor that serves as a central source of maternal satisfaction and identity. Stimulation refers to the belief that parents must actively provide cognitive stimulation to support children's optimal development. Challenging refers to the view of mothering as a demanding and exhausting task that requires enormous effort and devotion. Child-centered reflects the belief that children's needs should be the primary focus for parents.

As parenting beliefs take shape in sociocultural contexts, parents tend to endorse specific dimensions of IM beliefs that align with their cultural values and norms (Mollborn and Billingsley, 2025; Gauthier et al., 2021). For instance, French mothers endorsed child-centrism and stimulation sub-beliefs of intensive parenting but were less supportive of sacrifice and challenging sub-beliefs (Loyal et al., 2017). Israeli middle-class parents typically adhere to one of the two “folk models” of intensive parenting: child-centeredness and stimulation (Klimor Maman et al., 2024). These findings indicate that parents may adhere to multiple facets of IM beliefs differently depending on their sociocultural backgrounds. However, the majority of research on IM beliefs has been conducted in the Western cultural contexts.

In China, Confucian values underscore the central role of mothers in children's development and emphasize maternal dedication and self-sacrifice in supporting children's development. Moreover, education is widely accepted as as a primary pathway to upward social mobility, and the highly competitive educational system has intensified parents' anxieties regarding their children's future prospects. In socially mobile societies, such pressures prompt parents to be overly involved in children's everyday lives (Lubiewska et al., 2025). Given that the current extensive mothering literature has primarily sampled white and middle-class mothers in the Western cultures (Forbes et al., 2020), the present study focused on how Chinese mothers of preschool children from diverse socioeconomic backgrounds endorse distinct dimensions of IM beliefs.

### 1.2 Intensive mothering beliefs and children's development

IM beliefs constitute a multifaceted set of belief systems (Liss et al., 2013). Prior literature has yielded mixed results regarding the effects of multiple dimensions of IM beliefs on children's development outcomes. For example, Schiffrin et al. (2015) found that mothers' stronger endorsement of constant stimulation beliefs was associated with higher language skills and greater happiness among 2–5 years old preschool children, whereas essentialist beliefs were related to children's poorer motor skills. Egami (2024) sampled Japanese mothers of preschoolers and reported that maternal essentialist beliefs were associated with more externalizing behavior problems but fulfillment beliefs related to fewer externalizing problems. A recent study of Chinese preschoolers found that maternal essentialist and challenging beliefs were associated with poorer social-emotional competence in children (Li et al., 2024). Similarly, null effects of child-centered beliefs on children's motor, language and social development outcomes were reported in other studies sampling U.S. and Japanese preschoolers (Schiffrin et al., 2015; Egami, 2024).

Taken together, these findings suggest that multiple dimensions of IM beliefs are not uniformly beneficial or detrimental; instead, their effects vary across sub-dimensions. Stimulation and fulfillment beliefs may promote positive child outcomes, whereas essentialist and challenging beliefs are more consistently associated with risks in children's adjustment, and the effect of child-centered beliefs appears less consistent or negligible. However, the current literature on IM beliefs has mostly sampled white, middle-class women from relatively privileged families in the West (Schiffrin et al., 2015; Wall, 2010). Given the mixed effects of specific aspects of IM beliefs, the present study focused on how distinct facets of IM beliefs contribute to variances in Chinese preschoolers' internalizing and externalizing problems.

### 1.3 Overparenting behavior and children's development

According to Self-determination theory (Ryan and Deci, 2000), children's psychological needs for autonomy, competence, and relatedness are essential for optimal functioning. Parents' overparenting behaviors such as excessive protection and anticipatory problem solving for children may undermine children's sense of autonomy and competence, thereby contributing to children's maladjustment and behavioral problems (Segrin et al., 2015; Zhang and Ji, 2024). Attachment theory posits that a secure relational base provided by caregivers enables young children to explore their environment, regulate emotions, and develop social competence (Bowlby, 1977). Overparenting, characterized by excessive control and intrusion, may undermine this secure base by limiting children's opportunities to experience autonomy and manage age-appropriate challenges independently. Emerging empirical research has linked overparenting behavior to a range of negative outcomes, including increased narcissism, reliance on external academic motivation, emotional and behavioral problems, and difficulties in social and interpersonal relationships (Cui et al., 2022; Zhang and Ji, 2024). However, prior research has mostly sampled adolescents and emerging adults (Scharf et al., 2017; Segrin et al., 2012), and has given inadequate attention to young children.

Some scholars argue that although overparenting appears developmentally inappropriate during adolescence and emerging adulthood, its adverse effects may be less evident in early childhood (Yaffe et al., 2024). In contrast, other researchers have found that even in early childhood excessive involvement or protection contributed to children's behavior problems (Gulenc et al., 2018). For instance, Mäntymaa et al. (2004) reported that parents' intrusiveness predicted both internalizing and externalizing problems in 2-year-old children. Another empirical study with small samples (*n*=29) shows that overparenting behaviors positively predict U.S. school-age children's internalizing problems including anxiety, depression and low self-esteem (Schmidinger, 2020). Ruiz-Ortiz et al. (2024) found that paternal anticipatory problem solving practices positively related to Spanish elementary school children's school problems. The only research sampling Chinese preschoolers identified overparenting behavior as a significant predictor of children's social shyness (Zhang et al., 2024). Although research on Chinese over parenting remains scarce, several relevant studies implied the detrimental effects of over-protection and over control on young children's social-emotional adjustment. For example, Jian et al. (2025) found that maternal overprotective practices significantly predicted their children's anxiety levels and Sun et al. (2023) showed that maternal overcontrol significantly predicted 3–4 years old Chinese preschoolers' internalizing problems (i.e., fearfulness, anxious, and depression). As most studies on overparenting have been conducted in the Western contexts, the present study attempted to examine the relationships between maternal overparenting behaviors and preschool children's behavior problems in the Chinese context.

### 1.4 The indirect effect of intensive mothering beliefs via overparenting behavior

The parenting cognition-behavior-child adjustment model posits that parents' beliefs and values shape their daily care-giving practices, which in turn influence children's adjustment (Bornstein et al., 2018). Within this framework, parenting beliefs are not assumed to affect child adjustment directly but function indirectly through specific parenting behaviors. Empirical evidence suggests that parental IM beliefs are associated with greater parental involvement and monitoring in parent-child interactions (Lamprianidou et al., 2025) and positively predict maternal preemptive problem solving behaviors (Schiffrin et al., 2015). These findings suggest that when IM beliefs are enacted to an extreme degree, they may manifest as over parenting behaviors, including over control, excessive assistance, and overprotection (Turner et al., 2023).

Self-determination theory posits that children's psychological functioning is supported by the satisfaction of basic needs for autonomy, competence, and relatedness (Ryan and Deci, 2000). Prior research has shown that autonomy-thwarting and psychologically controlling parenting are associated with higher levels of anxiety, emotional dysregulation, and behavior problems in children (Jian et al., 2025; Sun et al., 2023). Parenting practices characterized by excessive control or intrusion may inadvertently constrain opportunities for independent exploration and mastery, thereby frustrating children's autonomy and competence needs. Also, empirical studies identified over parenting behavior as a significant predictor of children behavior problems (Schmidinger, 2020; Ruiz-Ortiz et al., 2024). Thus, consistent with cognition-behavior models of parenting, over parenting behavior may serve as a proximal mechanism through which IM beliefs may affect children's adjustment outcomes. However, this potential mediating pathway has received little empirical attention. Recent research suggests that IM beliefs have negligible direct effects on young children's social competence but affect child outcomes indirectly through negative parenting practices (Egami, 2024). In contrast, Schiffrin and associates failed to identify the mediating role of over parenting practice (i.e., anticipatory problem solving) in IM beliefs and preschool children's motor, language and wellbeing outcomes (2015). Given the limited research on the mediating pathway, further studies are needed to test whether over parenting behaviors significantly account for the associations between IM beliefs and children's development outcomes.

### 1.5 The present study

IM beliefs are prevalent in the contemporary society, however, parents' endorsement of intensive mothering beliefs varies across races and countries (Long et al., 2021). The majority studies have focused on white, middle-class mothers from Western countries (Faircloth, 2014), yet less is known about how IM beliefs operate across more diverse socioeconomic and cultural contexts. Cultural relativistic perspective (Deater-Deckard and Dodge, 1997) posits that the effects of parenting beliefs and practices are not universal but vary across cultural contexts. In the Chinese culture, mothers have traditionally been perceived as primarily responsible for their children's healthy development (Liu and Xu, 2024) and they tend to base their personal worth on children's achievement and success (Ng et al., 2014). Given that intensive parenting is culturally normative in the Chinese context (Doepke and Zilibotti, 2019), there is a necessity to explore how multiple aspects of IM beliefs would relate to preschool children's internalizing and externalizing behavior problems.

Although previous research has examined the links between intensive parenting ideologies, maternal wellbeing and child adjustment, less is known about the behavioral pathways through which these beliefs influence young children's social-emotional development. Drawing on the parenting cognition-behavior-child adjustment framework and self-determination theory, the present study proposes that maternal IM beliefs may be reflected in concrete parenting practices, specifically overparenting behaviors, which in turn are associated with children's behavioral outcomes. The research questions guiding this study are: 1. What are the relationships between mothers' IM beliefs and Chinese preschoolers' internalizing and externalizing problems? 2. How do intensive mothering beliefs and maternal over parenting behavior jointly relate to Chinese preschoolers' internalizing and externalizing behavior problems? Based on prior literature, this study proposed the following hypotheses:

H1: Higher levels of essentialist, challenging, and child-centered IM beliefs would relate to higher levels of internalizing and externalizing problems in Chinese preschoolers.
H2: Higher levels of fulfillment and stimulation IM beliefs would relate to lower levels of internalizing and externalizing problems in Chinese preschoolers.
H3: Mothers' IM beliefs would be indirectly associated with Chinese preschoolers' internalizing and externalizing problems via maternal overparenting behavior.

## 2 Methodology

### 2.1 Context and participants

A stratified cluster sampling method was used to recruit mothers and their preschool-age children. Specifically, a total of 1,025 mothers were recruited from eight preschools in Shanghai, China, including two “model” preschools, three “first-class” preschools, and three “second-class” preschools. Preschools in Shanghai provide services for 3 to 6 years old children. Generally, “model” and “first-class” preschools enroll more children from higher socioeconomic status (SES) families, while “second-class” preschools have more children from lower-SES family background. The mother participants ranged in age from 25 to 48 years (*Mean* = 36.42, *SD* =3.42). Their children had a mean age of 4.94 years (*SD* = 0.72) and slightly more than half (52.2%) were boys. The majority of families were intact (97.5%), and a substantial proportion (81.3%) reported grandparental involvement in child-rearing. Most mothers (86.8%) were employed either full-time or part-time. In terms of monthly family income, 7.1% families earned less than *RMB* 8,000 per month, 12.9% earned between *RMB* 8,000-15,000, 20% between *RMB* 15,000–20,000, 42% between *RMB* 20,001–50,000, and 18% reported monthly earning exceeding *RMB* 50,000. In terms of educational attainment, 5% of mothers had less than a high school education, 18.4% held a three-year college diploma, 59% held a bachelor's degree, and 17.6% had a master's degree or higher.

### 2.2 Measures

#### 2.2.1 Intensive mothering beliefs

The Intensive Parenting Attitude Questionnaire (IPAQ; Liss et al., 2013) was used to measure mothers' endorsement of intensive mothering beliefs. The IPAQ is composed of five dimensions based on 25 items: essentialism, fulfillment, challenging, stimulation, and child-centered. The dimension of essentialism (8 items) measures respondents' belief that females are better at parenting than males. The dimension of fulfillment (4 items) measures mothers' endorsement of the belief that a parent's happiness is derived primarily from their children and mothering is a fulfilling task. The dimension of stimulation (4 items) asked mothers questions about their attitudes about parents' responsibility for the cognitive stimulation of their children. The dimension of challenging (6 items) examined mothers' belief that parenting is a difficult and demanding task. The dimension of child-centered (3 items) captures mothers' agreement that children should be the center of parents' attention and parents should put their children's needs above their own. Respondents were asked to report the extent to which they agreed or disagreed on a six-point scale (1=strongly disagree, to 6=strongly agree). An average score was calculated for each subscale with higher scores representing stronger endorsement of intensive mothering beliefs. Cronbach's alphas for the five dimensions were 0.73 (Essentialism), 0.75 (Fulfillment), 0.56 (Stimulation), 0.67 (Challenging), and 0.68 (Child-centered). The dimension of stimulation was eliminated from further analysis because of the low reliability coefficient.

#### 2.2.2 Overparenting behavior

Maternal over parenting behavior was assessed using the anticipatory problem solving subscale of the Over parenting Scale (OPS, Segrin et al., 2012). The original instrument was developed for adolescents and adult populations and consisted of four subscales: anticipatory problem solving, advice/affect management, self-direction and tangible assistance. In the present study, the anticipatory problem solving subscale was used as an indicator of maternal over parenting as prior research suggests that this behavior is particularly appropriate for assessing over parenting in early childhood and is a strong predictor of children's psychosocial development outcomes (Scharf et al., 2017; Schiffrin et al., 2015). The other three subscales either describe behaviors that are not relevant to early childhood or reflect behaviors that are normative for this developmental stage. The anticipatory problem solving subscale measures the degrees to which mothers intervene preemptively in children's daily lives, anticipate potential difficulties and remove obstacles before children even encounter them. This subscale consists of 12 items (e.g., “I try to help my child steer clear of any troubles that s/he might encounter in the world.”) based on a 5-point Likert-type scale ranging from 1 (strongly disagree) to 5 (strongly agree). Items in the subscale were averaged to obtain a score to represent mothers' over parenting behavior. Higher scores indicate that mothers display higher over parenting behavior. The reliability of this subscale was 0.88, supporting its reliability for use in a preschool sample.

#### 2.2.3 Children's internalizing and externalizing problems

Children's internalizing and externalizing behavior problems were assessed using the Strengths and Difficulties Questionnaire (SDQ; Goodman et al., 2010). The SDQ is a widely used parent-report questionnaire designed to evaluate children's social-emotional and behavior functioning. The 25-item instrument consists of 5 dimensions: emotional symptoms, conduct problems, hyperactivity/inattention, peer relationship problems, and pro social behavior. Mothers were asked to make responses based on their children's behavior in the last 6 months. Responses were reported on a 3-point Likert scale ranging from 0 (not true) to 2 (certainly true). As recommended by Goodman et al. (2010), it is appropriate to use broader internalizing and externalizing SDQ subscales with low-risk samples as there may be no salient differences in specific subscales among low-risk populations. Following the scoring guidelines of the SDQ, the internalizing score was calculated by summing the emotional and peer problems sub-scales, while the externalizing score was derived from the sum of the conduct problems and hyperactivity/inattention subscales. Cronbach's alphas for children's internalizing and externalizing behavior problems were 0.71 and 0.72 respectively.

### 2.3 Data collection

At first, the IRB permission was obtained from the university, and then principals of the target preschools in Shanghai were contacted for the possibility of collecting data in their schools. Eventually, eight preschools were involved in the present study. Mothers were informed about the purpose and procedures of the study and if they agreed to participate in the study, they provided the informed consent forms. Mother participants were asked to fill out the questionnaire including demographic information (e.g., child gender, child age, family income), intensive mothering beliefs, over parenting behaviors and assessed their preschool children's social-emotional and behavioral competence using a widely used parent-report instrument.

### 2.4 Data analysis

At first, descriptive statistics and correlation analyses for the study variables were conducted using SPSS 26.0 to identify potential control variables. Second, two hierarchical multiple regression analyses were performed to examine the associations between the various dimensions of IM beliefs and preschoolers' internalizing and externalizing problems. In each model, children's internalizing and externalizing problem scores were regressed on multiple dimensions of IM beliefs while controlling for demographic variables. The dimensions of IM beliefs that emerged as significant predictors were retained for further analyses. Third, to test the indirect relationship with children's behavior problems via maternal overparenting behavior, structural equation modeling (SEM) analysis was conducted using Amos 22.0, with IM beliefs as predictors, overparenting behavior as the mediator, and children's internalizing and externalizing problems as the outcome variables. In the SEM analysis, the three significant dimensions (i.e., essentialism, challenging, and child-centered) were modeled as indicators of a latent intensive mothering construct rather than as separate predictors, thereby preserving its multidimensionality while enabling a more parsimonious test of the mediation model. To determine the adequacy of the model fit, the following goodness-of-fit indexes were used to test the model fit: non-significant Chi-square (χ^2^) value (*p* > 0.05), Chi-square ratios between one and three, the Comparative Fit Index (CFI) > 0.90, the Tucker-Lewis Index (TLI) > 0.90, Root Mean Square Error of Approximation (RMSEA) <0.06, and Standardized Root Mean Square Residual (SRMR) <0.08 (Kline, 2005).

## 3 Results

### 3.1 Common method bias testing

In this study, all the data were collected through mothers' self-report survey. To test the common method bias, a confirmatory factor analysis (CFA) was conducted using Harman's one-factor test (Podsakoff et al., 2003). The results showed that the first factor accounted for 15.66% of the total variance, which is well below the critical threshold of 40%. This indicates that common method bias was not a significant concern in the present study.

### 3.2 Descriptive analysis

Means, standard deviations, and correlations for the main variables were presented in Table 1. On a 6-point scale, mothers reported relatively high levels of challenging beliefs (*M* = 5.32, *SD* = 1.13) and fulfillment (*M* = 4.79, *SD* = 1.03), indicating strong endorsement of the demanding yet rewarding nature of intensive mothering. In contrast, essentialism (*M* = 3.96, *SD* = 1.27) and child-centeredness (*M* = 3.76, *SD* = 1.00) were endorsed at moderate levels. Over parenting behavior, measured on a 5-point scale, was also reported at a moderate level (*M* = 3.09, *SD* = 0.65).

**Table 1:** Descriptive statistics and zero-order correlations for all variables.

Variable	2	3	4	5	6	7	8	9	10	11
1.Child's gender	−0.04	0.02	−0.04	0.04	−0.04	−0.02	0.05	0.04	−0.05	−0.06^*^
2.Only child	–	−0.05	0.05	0.03	0.06^*^	0.01	0.03	−0.02	−0.03	−0.13^***^
3.Mother education		–	0.31^***^	−0.13^***^	−0.05	−0.08^*^	−0.10^**^	0.01	−0.07^*^	−0.12^***^
4.Family income			–	−0.09^**^	−0.03	−0.13^***^	−0.13^***^	−0.10^**^	−0.19^***^	−0.16^***^
5.Essentialism				–	0.19^***^	0.46^***^	0.42^***^	0.31^***^	0.19^***^	0.19^***^
6.Fulfillment					–	0.36^***^	0.40^***^	0.26^***^	0.05	0.02
7.Challenging						–	0.45^***^	0.35^***^	0.21^***^	0.17^***^
8.Child-centered							–	0.48^***^	0.20^***^	0.12^***^
9.Overparenting								–	0.29^***^	0.20^***^
10.Internalizing									–	0.61^***^
11.Externalizing										–
*Range*				1–6	1–6	1–6	1–6	1–5	0–20	0–20
*Mean*	–	–	–	3.96	4.79	5.32	3.76	3.09	3.96	4.86
*SD*	–	–	–	1.27	1.03	1.13	1.00	0.65	3.06	3.15

*n* = 1025. ^*^p < 0.05; ^**^p < 0.01; ^***^p < 0.001.

Correlational analyses revealed several significant associations. As shown in Table 1, children's gender, only child status, family income and maternal educational level were significantly linked with children's internalizing and externalizing problems. Therefore, they were included as control variables in further analysis. As for the main variables of the study, three dimensions of IM beliefs (i.e., esentialism, challenging, and child-centered) were positively related to preschoolers' internalizing and externalizing problems, *rs* = 0.12-0.21, *ps* < 0.001. Over parenting behavior was positively related to all four dimensions of IM beliefs (*rs* = 0.26–0.48, *ps* < 0.001) and to children's internalizing problems (*r* = 0.29, *p* < 0.001) and externalizing problems (*r* = 0.20, *p* < 0.001).

### 3.3 Intensive mothering beliefs and preschoolers' behavior problems

Two hierarchical multiple regressions were conducted to examine the associations between maternal endorsement of IM beliefs and preschoolers' internalizing and externalizing problems while controlling for demographic variables (see Table 2). For internalizing problems, the demographic variables in step 1 accounted for 3.9% of the variance, Δ*R*^2^ = 0.039, [*F*(4, 1020) = 10.39, *p* < 0.001]. Specifically, child's gender (β = −0.06, *p* < 0.05) and family income (β = −0.18, *p* < 0.001) significantly predicted internalizing problems, with boys and children from lower-income families showing higher levels of internalizing problems. When intensive parenting beliefs were added in Step 2, an additional 5.3% of the variance was explained, Δ*R*^2^ = 0.053, [*F*_(4.1016)_ = 14.96, *p* < 0.001]. Three dimensions emerged as significant individual predictors: essentialism (β = 0.08, *p* < 0.05), challenging (β = 0.11, *p* < 0.01), and child-centered beliefs (β = 0.12, *p* < 0.001). The fulfillment dimension of IM beliefs was not a significant predictor (*p*>0.05).

**Table 2:** Sequential multiple regressions of behavior problems on intensive mothering beliefs.

Variable	*B*	*SE B*	β	*ΔR^2^*	*F*	*t*	*P*
Internalizing problems							
Step 1				0.039	10.39^***^		0.00
Child's gender	−0.04	0.02	−0.06			−2.03^*^	0.04
Only child	−0.02	0.02	−0.02			−0.77	0.44
Mother education	−0.01	0.01	−0.02			−0.58	0.56
Family income	−0.04	0.01	−0.18			−5.62^***^	0.00
Step 2				0.053	14.96^***^		0.00
Essentialism	0.03	0.01	0.08			2.39^*^	0.02
Fulfillment	−0.02	0.01	−0.06			−1.73	0.08
Challenging	0.03	0.01	0.11			3.01^**^	0.01
Child-centered	0.03	0.01	0.12			3.38^***^	0.01
Externalizing problems							
Step 1				0.053	14.29^***^		0.00
Child's gender	−0.04	0.02	−0.07			−2.25^*^	0.02
Only child	−0.08	0.02	−0.13			−4.13^***^	0.00
Mother education	−0.04	0.01	−0.09			−2.74^**^	0.01
Family income	−0.03	0.01	−0.14			−4.20^***^	0.00
Step 2				0.038	10.60^***^		0.00
Essentialism	0.04	0.01	0.12			3.44^**^	0.01
Fulfillment	−0.02	0.01	−0.06			−1.80	0.07
Challenging	0.03	0.01	0.10			2.73^**^	0.01
Child-centered	0.01	0.01	0.04			1.01	0.31

*n* = 1,025 ^*^p < 0.05; ^**^p < 0.01;^***^p < 0.001.

For externalizing problems, demographic controls in Step 1 accounted for 5.3% of the variance, Δ*R*^2^ = 0.053, [*F*(4, 1020) = 14.29, *p* < 0.001. Child's gender (β = −0.07, *p* < 0.05), only-child status (β = −0.13, *p* < 0.001), maternal education (β = −0.09, *p* < 0.01), and family income (β = −0.14, *p* < 0.001) were all significant predictors, indicating that boys, only child, children of less-educated mothers, and children from lower-income families were at greater risk of externalizing problems. The addition of IM beliefs in Step 2 explained an additional 3.8% of variance, Δ*R*^2^ = 0.038, [*F*(4, 1016) = 10.60, *p* < 0.001]. Both essentialist (β = 0.12, *p* < 0.01) and challenging beliefs (β = 0.10, *p* < 0.01) significantly predicted higher levels of externalizing problems. Fulfillment and child-centered beliefs were not significant predictors (*p* > 0.05).

### 3.4 The indirect effect of intensive mothering beliefs via overparenting behavior

**Figure 1:**
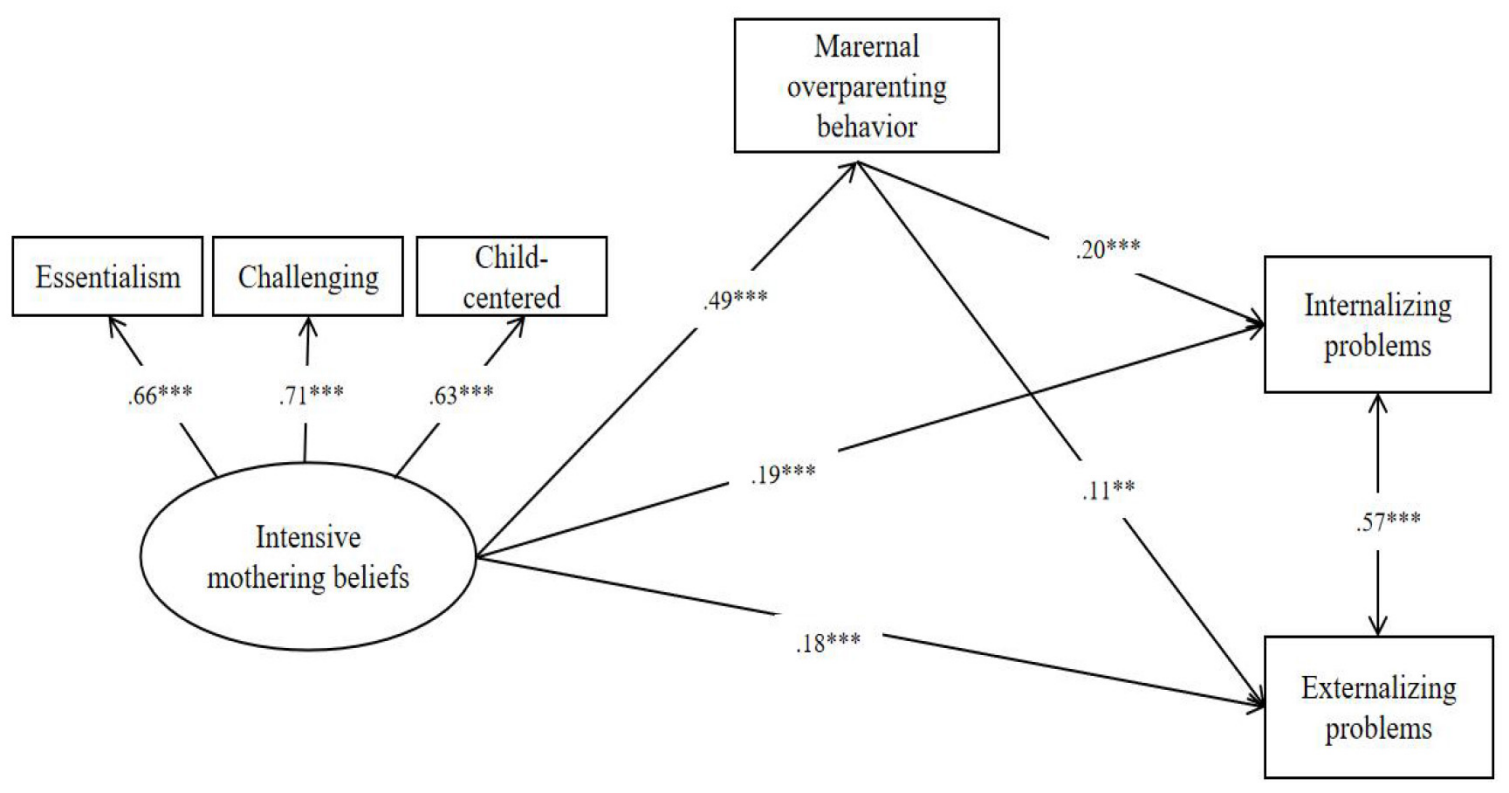
The direct and indirect effects of intensive mothering beliefs via overparenting behavior. *N* = 1,025. **p < 0.01, ***p < 0.001.

Structural equation modeling was conducted to examine the direct and indirect associations among mothers' IM beliefs, maternal overparenting behavior, and preschoolers' behavioral problems. The model demonstrated a good fit to the data, χ^2^*/df* = 1.52, *p* = 0.18, CFI = 0.99., TLI = 0.99, IFI = 0.99, RMSEA = 0.023 (90% CI = 0.00−0.05), SRMR = 0.012. Standardized parameter estimates for the model were presented in Figure 1.

As shown in Figure 1, mothers' IM beliefs were positively associated with maternal over parenting behavior (β = 0.49, *p* < 0.001), indicating that stronger endorsement of intensive mothering ideology was related to higher levels of over parenting practices. With respect to child outcomes, IM beliefs were directly associated with higher levels of preschoolers' internalizing problems (β = 0.19, *p* < 0.001) and externalizing problems (β =0.18, *p* < 0.001). Also, maternal overparenting behavior was positively associated with both internalizing (β = 0.20, *p* < 0.001) and externalizing problems (β = 0.11, *p* < 0.01).

Indirect effects were tested to examine whether overparenting behavior statistically accounted for the associations between IM beliefs and preschoolers' behavioral problems. Results indicated that the indirect effect of IM beliefs on internalizing problems via over parenting behavior was statistically significant (β_*indirect*_ = 0.10, *p* < 0.001). Similarly, the indirect effect of IM beliefs on externalizing problems through over parenting behavior was also statistically significant (β_*indirect*_ = 0.05, *p* < 0.01). Maternal over parenting behavior accounted for 34% of the effect of IM beliefs on internalizing problems and 23% of the effect on internalizing problems. These results indicate that mothers' IM beliefs were indirectly associated with Chinese preschoolers' internalizing and externalizing problems via maternal over parenting behavior.

## 4 Discussion

### 4.1 Intensive mothering beliefs and preschoolers' behavior problems

Controlling for family and child demographics, multiple regression analyses indicate that multiple dimensions of intensive mothering (IM) beliefs, particularly essentialist, challenging, and child-centered beliefs, were significantly associated with higher internalizing problems in children. Also, essentialist and challenging IM beliefs significantly related to higher externalizing problems in children. These findings are consistent with an emerging body of literature suggesting that intensive parenting ideologies may have unintended developmental costs (Yerkes et al., 2021). For instance, Schiffrin et al. (2015) found that greater endorsement of essentialism beliefs related to poorer motor skills in U.S. preschool children and Egami (2024) reported that mothers' essentialist beliefs related to more externalizing behavior problems in Japanese preschoolers. One possible explanation for the negative effects of essentialist and challenging beliefs is the heightened parenting stress and anxiety that often arise from IM beliefs (Novoa et al., 2025; Kim and Kerr, 2024). Such increased parenting stress, in turn, has been shown to predict poorer social-emotional competence and executive functioning in children (Li et al., 2024; McGregor, 2022). When mothers perceive parenting as overwhelmingly exhausting and challenging, they are more vulnerable to emotional fatigue and diminished patience, which would in turn impair parent-child interactions and increase children's risks of social-emotional maladjustment (Crnic et al., 2005; Rizzo et al., 2013). Another explanation might be that mothers in this study were largely employed in the workplace. When combined with employment responsibilities, essentialist and challenging beliefs may exacerbate stress, guilt, and burnout, as mothers struggle to reconcile professional obligations with the perceived responsibility for child-rearing.

In addition, the present study showed that child-centered beliefs were associated with higher internalizing behavior problems in preschoolers. Parents who endorse child-centered beliefs often prioritize their children's needs above all else, which can manifest as heightened control and over-involvement in daily activities (Schiffrin et al., 2014). Such intensive parental engagement may inadvertently constrain children's autonomy and limit opportunities for independent problem-solving, thereby increasing their risks of behavior problems (Wall, 2010). This finding contrasts with much prior research that has generally reported non-significant relationships between child-centered beliefs and preschoolers' development outcomes (Egami, 2024; Schiffrin et al., 2015). One explanation for this inconsistency is that the effects of child-centered beliefs may depend on how they are enacted and experienced by mothers. When child-centrism manifests as active, intrinsically rewarding engagement in caregiving activities, mothers may experience positive affect, a sense of meaning, and satisfaction (Ashton-James et al., 2013). Under these conditions, children may benefit from enriched interactions and supportive environments provided by intensive mothers. However, when child-centered beliefs are perceived as prescriptive high standards or enduring obligations for care-giving, mothers may experience sustained stress, depressive symptoms, and lower life satisfaction (Rizzo et al., 2013). In this context, maternal stress may lead to more controlling or intrusive parenting behaviors and increase children's risks of internalizing problems. Moreover, the positive association between child-centered IM beliefs and children's internalizing problems can be accounted for by traditional Chinese cultural values. Rooted in Confucian values such as “*Mu Yi Zi Gui*” (a mother's worth is reflected in her children) and “*Jiao Zi You Fang*” (competent child-rearing as a moral duty), children's achievements and behaviors are often seen as a reflection of maternal worth and family competence. In this context, child-centered IM beliefs may prompt mothers to become highly and emotionally invested in their children and impose pressure for their children to perform well. For preschool children, such pressure may be internalized as anxiety, fear of disapproval, or a strong need to meet parental standards, thereby increasing their vulnerability to internalizing problems.

### 4.2 The indirect effect of intensive mothering beliefs via overparenting behavior

The structural equation modeling analysis indicated that mothers who more strongly endorsed essentialist, challenging, and child-centered IM beliefs were more likely to engage in over parenting behavior. These findings align with a prior empirical study on U.S. preschoolers, which found that mothers' endorsement of essentialist and child-centered beliefs were positively associated with parents' anticipatory problem solving behaviors (Schiffrin et al., 2015). This pattern suggests that intensive mothering beliefs often translate into excessive involvement and control during early childhood. According to the Family Stress Model (Conger et al., 2010), intensive mothering beliefs may be understood as chronic cognitive stressors that heighten mothers' perceived responsibility for children's development and increase sensitivity to potential risks for their children. Such belief system can lead mothers to engage in overprotective, controlling, and preemptive problem-solving behaviors by intervening in children's activities to minimize perceived threats and ensure optimal outcomes. In addition, empirical research shows that strong endorsement of IM beliefs is associated with increased parenting guilt, burnout and distress (Kim and Kerr, 2024; McGregor and Shannon, 2025). These maternal psychological strains may lead mothers to adopt more controlling and intrusive parenting behaviors, which can increase the risk of behavioral problems in children.

Importantly, results showed that maternal over parenting behavior was also directly linked to higher levels of internalizing and externalizing problems in preschoolers. These findings are consistent with prior studies that identified the negative effects of over parenting on children's social-emotional outcomes. For instance, Schmidinger (2020) found that parents' over parenting behaviors significantly predicted U.S. school-age children's externalizing problems. Ruiz-Ortiz et al. (2024) found that paternal anticipatory problem solving behavior positively related to Spanish elementary school children's school problems. Also, the results in the present study echoes a recent study sampling Chinese preschoolers which found over parenting behavior as a significant predictor of children's social shyness (Zhang et al., 2024). Self-determination theory posits that when children's psychological needs for autonomy and competence are frustrated, their social-emotional development may be adversely affected (Ryan and Deci, 2000). When parents consistently intervene to preemptively resolve problems for their children, they may inadvertently convey negative messages to their children that they are not competent or incapable of managing challenges independently (Hong and Cui, 2023). These experiences limit children's opportunities to practice autonomy, self-regulation, and independent problem-solving skills (Jian et al., 2025; Sun et al., 2023) and increase their vulnerability to internalizing symptoms such as anxiety, withdrawal and externalizing problems such as frustration and defiance (Jiang et al., 2023).

Moreover, the present study provides empirical evidence that overparenting behavior partially explained the associations between IM beliefs and preschoolers' internalizing and externalizing behavior problems. This finding is consistent with the current literature that identified overparenting as a proximal behavioral pathway linking parenting beliefs with child outcomes. For instance, Egami (2024) found that maternal intensive parenting attitudes influenced children's social competence through parenting behaviors such as excessive involvement and monitoring and Ruiz-Ortiz et al. (2024) identified overparenting behavior as a mediator linking parental characteristics (e.g., parental education level) with children's school problems. Maternal beliefs in essentialism, challenging and child-centrism may lead mothers to engage in over-involvement and overprotective behaviors (Egami, 2024; Schiffrin et al., 2015), which in turn contribute to increased levels of both internalizing and externalizing problems in children (Locke, 2014; Schiffrin et al., 2014). In the Chinese cultural context, these patterns are exemplified by the phenomenon of “Tiger Mothers” who demonstrate intensive involvement in children's lives, exercise strict control, and closely supervise children's daily activities (Zhang and Wang, 2025). Although motivated by a desire to foster skill development and academic success, such over-involved and controlling parenting can inadvertently restrict children's autonomy, reduce opportunities for independent exploration, and increase the risk of both internalizing and externalizing problems (Jiang et al., 2023; Sun et al., 2023).

## 5 Implications

The present study found that intensive mothering beliefs accounted for variances in preschoolers' behavioral problems and maternal overparenting behavior partially mediated this relationship. Although the effect sizes were statistically significant, they were relatively small and should be interpreted with caution. Despite this, the findings offer several implications for research and practice. First, mothers' stronger endorsement of “essentialist”, “challenging” and “child-centered” beliefs related to higher behavioral problems in preschoolers. These results support the growing body of literature about the potential negative effects of IM beliefs. It is necessary to reevaluate the cultural norms that position intensive mothering as the standard of “good” parenting and inform parents about the potential unintended consequences of intensive mothering beliefs. Second, the present study provides preliminary evidence that maternal overparenting behavior serve as a potential mediator linking IM beliefs and child outcomes. Although the indirect effect was modest, it suggests that IM beliefs may influence children's behavioral adjustment through overparenting practices, such as anticipatory problem solving and premature intervention in children's activities. Schools and community organizations can offer workshops, seminars, and parent-child activities that promote balanced parenting, emphasizing warmth, responsiveness and sensitive guidance while also encouraging age-appropriate independence. Early childhood educators can guide parents on providing children with opportunities to develop self-regulation, decision-making and problem-solving skills. Parent education programs may benefit from encouraging developmentally appropriate autonomy support and helping parents reflect on when and how to step back from excessive involvement in children's daily activities. Such efforts may help mitigate potential risks associated with IM beliefs and overparenting behavior and promote healthier social-emotional and behavioral adjustment in preschool children.

## 6 Limitations and suggestions for further research

Despite its contributions, several limitations of this study should be acknowledged. First, the data were collected from only one city in China, Shanghai, which limits the generalizability of the findings to a larger population of the whole China. The city where the study was conducted represents a relatively developed and competitive social context, where IM beliefs and overparenting practice may be more salient compared to economically underdeveloped areas of China. Future research should include more diverse samples across different geographic and socioeconomic contexts in China to capture variations in IM beliefs, practices, and their associations with children's behavioral outcomes. Second, the study relied exclusively on maternal self-reports of IM beliefs, overparenting behaviors, and children's behavior problems. Such reports may be influenced by social desirability or mothers' subjective perceptions, raising concerns about potential response bias. The inclusion of multi-informant data, such as teacher reports or direct behavioral observations, could provide a more comprehensive assessment of child adjustment and reduce potential bias associated with parent-reported measures. Third, the present study did not separately assess paternal and maternal IM beliefs and overparenting behaviors. Fathers and mothers may perceive IM beliefs differently and have different effects on preschool children's social-emotional and behavioral outcomes. Fourth, as the the Overparenting Scale was originally designed for adolescents and emerging adults, this study exclusively used the anticipatory problem solving subscale as an indicator of maternal overparenting behavior to enhance developmental appropriateness for preschool children. Hence, certain aspects of overparenting in early childhood may not be fully captured. Future research is encouraged to develop and validate overparenting instruments specifically tailored to early childhood contexts.

## 7 Conclusion

Although the dominant ideology of intensive mothering is widely accepted, empirical evidence on its beneficial effects has been rather limited. This study demonstrates that mothers' endorsement of essentialist, challenging, and child-centeredness IM beliefs is associated with higher maternal overparenting behaviors, which in turn increase preschoolers' vulnerability to behavioral problems. These findings challenge the culturally reinforced expectations of constant maternal responsibility and child-centered motherhood. Family education programs should encourage mothers to critically reflect on intensive mothering norms and adopt developmentally appropriate, autonomy-supportive parenting practices to promote children's social-emotional development.

## Data Availability

The raw data supporting the conclusions of this article will be made available by the authors, without undue reservation.
